# Mucous Membrane of the Mouth

**Published:** 1852-01

**Authors:** W. R. Handy


### AMERICAN JOURNAL

OF

### DENTAL SCIENCE.

Vol. II. NEW SERIES—JANUARY, 1852. NO. 2.

### ARTICLE I.

*Mucous Membrane of the Mouth.* By Professor W. R. Handy, M. D.

If there be one tissue of the body of higher relative importance to the practical dentist, than that of any other, it most assuredly must be that of the mucous membrane of the mouth. For, with this membrane is associated every thing of interest and utility connected with his profession. And by its relations with the rest of the tissues, comprehends both the *science* and the *art* of dentistry—the *science*, in demanding as an essential prerequisite, that it shall be studied, and thoroughly studied, in its anatomy, physiology, and pathology, as being the membrane which gives existence to the teeth, forms the foundation of their structure, and involves their several functions and diseases. And in the art, which simply embodies the proper application of this knowledge, in all the practical details relating to the proper management of the teeth.

"I cannot forbear," says Mr. Nasmyth, "from endeavoring to impress upon the minds of my readers, that such a knowledge forms the basis of sound surgical practice, and that without it, the surgeon would be incompetent to perform the most simple operations on the teeth, with a proper regard for the safety of his patient. The physiology of the teeth teaches us the cause of their shape and arrangement, their admirable adaptation to the office which they fulfil, and the manner in which their function is effected."

The mucous membrane further forms one of the two great divisions of the germinal membrane, the other, being the serous, whose combination, we are told, constitutes the human being in its earliest period of existence—the mucous division forms the minor portion, from whence spring all the organs of nutrition, and belong, in the language of Bichat, to organic or vegetable life, while the outer, or serous portion, forming the organs of locomotion, speech and sense, constitute animal life.

The mucous membrane, in the department of semeiology, furnishes one of the most important keys to unlocking the general condition of the system, as well as reporting every special departure in its own composition, whether that departure be in the color and density of its structure, or in the quality of the fluids of its function, thus furnishing, as it were, a kind of measurement, of the reciprocal influence exerted by this membrane and the system at large, and thereby enabling the dentist to give a practical value to such information, so practical as to tell him the time *when* he should operate, and so valuable as to make him avoid causing any unnecessary suffering to his patient, if no operation be demanded.

This membrane is also the great seat and recipient in the application of foreign agencies to the body, whether these agencies relate to its nutrition on the one hand, in supplying the various materials of nourishment, or to its medication on the other, in being the medium through which, is most generally introduced the different articles of the materia medica.

The mucous membrane in its full development in the teeth, is further turned to practical account by Mr. Sanders, in showing that their successive appearance is a more trustworthy test of *age*, than that of height, or even the testimony of parents, where their cupidity tempts them to sacrifice the health of their children and the truth, by putting them to work in the factories at an age too young—entirely unsuited to their physical strength, as well as in direct violation of law.

This membrane, also, as represented in the teeth, whose variety of structure the microscope has established, as existing in the various classes of animals, is thought to furnish the most natural foundation for a correct classification of the animal kingdom. And still further to indicate, agreeably to Mr. Nasmyth, "the progressive improvement of the human race." And, lastly, the multitude of diseases to which this membrane is subject, viz. schirrus, cancer, polypus; serous, mucous and bloody fluxes, with inflammation in all its varieties, &c., &c., render it a tissue of extreme interest.

Such are some of the considerations as bearing upon the *importance* of the mucous membrane, which we now propose to examine somewhat in detail, though in as brief a compass as possible, and as having a more especial reference to practical points in which the dentist as well as physician, is mainly concerned.

*Mucous Membrane*, (so called from the character of its secretion,) it is well known, chiefly occupies the interior of the body, and lines the whole of the alimentary canal, from the mouth to the anus, with the various glandular ducts which pour their fluids into it, also the respiratory canal, and the canals of the urinary and genital systems both of the male and female. It consequently enters as one of the fundamental elements in the great functions of digestion, respiration, urination and generation. It is also styled the *internal skin*, or tegument of the body, in contradistinction to the skin proper, or external tegument, which title seems to be fully justified, both by the continuity, as well as similarity of structure in the two divisions. At all the great outlets of the body, as those of the mouth, nose, anus, vagina, &c., &c., the mucus is traced as a continuous tissue with that of the skin, and further, the identity of the two is observed by the one being readily convertible into the other, as seen in prolapsus of the vagina, and rectum, where the mucus, by being exposed to the air, becomes changed into skin, and the skin, as in the axillar and between the nates by being kept moist and deprived of air, (as sometimes occurs in children, where cleanliness has not been observed,) assumes the character of mucous membrane. And as to similarity of structure, this will be noticed more particularly presently.

The comparative *extent* of the skin and mucous membrane, may be readily conceded in favor of the latter, when we consider for a moment its two great primary divisions, and but briefly follow the extent of surface which each covers. These divisions consist of the *gastro-pulmonary*, and *gento-urinary*—the former comprising the mucous membrane lining the mouth, pharynx, œsophagus, stomach, and intestines, with all their glandular ducts constituting the digestive apparatus, with that of the nose, larynx, trachea, and bronchia, ramifying in the lungs—forming the respiratory, while the latter extends from the kidneys, lining the uterus, bladder, and urethra, including the organs of urination with the vagina, uterus, and fallopian tubes of the female, and the several organs of the male, embracing those of generation.

Mucous membrane every where, presents the common character of a soft tissue, readily yielding to mechanical violence, easily destroyed by the action of chemical agents, and undergoing, with facility, the process of putrefaction.

The *thickness*, *consistency*, *color*, and *adhesiveness* of this membrane, varies at different points of its course, though all compatible with health, and in perfect harmony with the function which each separate part has to perform. As for instance, the thickness is found to be much greater in the alimentary canal, than in the urinary and genital organs, and greater in the duodenum, stomach, and rectum, than in any other part of the intestinal tube. The consistency equally varies, being greater at the pyloric than cardiac extremity of the stomach, greater in the lower portion of the jejuum and ileum, than in the stomach or duodenum, and much greater in the gums than either the lips, palate or cheeks. The color, if any thing, presents even a still greater diversity, for here the variation is not confined to the different parts of the mucous membrane, but the same part also may exhibit a difference of color, according to activity of function, and yet be in a state of health, as in the stomach, during digestion, the color is of a brighter red, than when this function is not going on, and is entirely due to the cause of the presence of a much larger quantity of blood, with greater activity of the circulation, than in the quiescent state, and yet both conditions healthy.

*Age* causes great variation of color. In the fetus it is seen of a deep rose color; in the adult, much paler, and in old age, presenting rather an ashy or grayish hue—in fact, the color seeming to be deep, or otherwise, according to the arterial development. Disease, the kind of death, and various substances introduced into the stomach, change, in greater or less degree, the color of the mucous membrane.

In acute disease, the color will be much deeper, than in chronic, where it will assume rather a palish aspect, from the poverty as well as diminution of blood. In sudden and violent death, as hanging, drowning, &c., the color is seen of a deep red; and substances such as nitrate of silver, logwood, lavender, &c., can each alter the color from its natural state; facts, all of which are highly interesting in a medico-legal point of view. In *structure*, the mucous membrane, though presenting peculiarities at different points, has, nevertheless, some characters which are common to the whole. This membrane is now generally admitted, by anatomists, to consist of three elements, viz. an epithelium, a basement, primary or papillary membrane, and an areolar tissue, containing the blood vessels and nerves.

The epithelium forms the outer layer, and is to mucous membrane, what the cuticle or epidermis is to the skin. It was thought, at one period, to be so partial, as only lining the mouth and œsophagus as far as the cardiac orifice of the stomach, but by the microscope, the epithelium is now satisfactorily traced throughout the whole of the alimentary tube.

It is found to be composed of cells, which, like the cuticle, are susceptible of rapid formation and of as rapid destruction, and consequently obeying the law of constant loss and renewal. These cells, though varying in their form in different parts, nevertheless perform the common office of protecting the mucous membrane from the contact of foreign bodies, whether dietetic or medicinal, and furnishing a smooth surface by the presence of the mucus, for the ready passage of alimentary material, and the various fluids of the several excretory ducts, without which provision, it does not seem hazarding too much to say, that for all the great practical purposes of health and life, the mucous membrane would not only be crippled, but wholly inoperative. The cells of the epithelium are arranged under three forms, viz. 1. The *tesselated*, or *pavement* epithelium. 2. The *cylindrical*, or *conical*, and 3. The *ciliated*.

The first variety is described as having its cells of various size, flattened, and either oval, roundish or polygonal, and are seen either in one layer or in many layers, placed one above the other. Each cell contains a central nucleus and nucleus corpuscle. This variety is found in the mouth, pharynx, œsophagus, and vagina, and is also seen lining serous and synovial membranes, blood and lymph vessels, and the cuticle likewise, is said to present the same plan of arrangement. The second variety is found to line the whole alimentary tube, from the cardiac orifice of the stomach to the anus—the principal gland ducts—the female urinary, and the male genito-urinary apparatus. Its cells, the microscope informs us, are of a cylindrical, columnar, or pyramidal shape, having their apices resting upon the basement membrane, while their bases approximating, form the free surface. The cylindrical cells are seen to be closely arranged, side by side ; containing, also, in each, a central nucleus and nucleolus. The third variety differ rom the second, simply in having the free extremity of its cylinders presenting little processes, termed cilia, and are found upon the mucous respiratory tract—the female organs of generation commencing at the neck of the uterus, and are also seen in the ventricles of the brain. These cilia are represented as in constant vibratory motion, and having their direction towards the outlets of the various canals they line, and designed, it is thought, to favor the motion of the fluids in their natural course, as for example, in the case of catarrh, to conduct the expectoration towards the outlets of the nostrils and mouth.*

*The second layer of the mucous membrane* is compared to the rete mucosum of the skin, and is regarded as the formation membrane of the epithelium, beneath which it is situated, and is called the *primary* or *basement* membrane. This membrane is structureless, and is considered by Mr. Carpenter, as forming an exception to the doctrine of Schwan, which makes all the tissues to have their origin from cells—he regarding its formation rather from "a layer of the plastic element."

*The third layer of mucous membrane* resembles the corium of the skin, though it is not so thick. It consists of areolar tissue, containing white and yellow fibres, also the blood vessels, and nerves, and gives to the mucous membrane its proper support and strength. A fourth element belonging to mucous membrane, consists of glands. These are found scattered throughout the whole mucous tissue of the alimentary tube, and are estimated by Dr. Horner to be upwards of forty-six millions. They are divided into three classes, viz. the follicles of Lieberkuhn, the glands of Brunner, and those of Peyer. The follicles are regarded as simple depressions in the mucous membrane, from whence the mucus is supposed to be furnished. But Dr. Horner regards these follicles of Lieberkuhn, as nothing more than the "meshes" in the veins, which his anatomical preparations present as cribriform in their appearance, and giving the follicular

* To Mr. Nasmyth the profession is much indebted for correct knowledge in reference to the structure of the epithelium. In his late microscopic researches, the scales of Leeuwenhoeck, or the squamous epithelium, as applied to the cuticle, as well as that lining the mouth, he describes as presenting four successive stages of development, viz. "1st. The promotion of nuclei and corpuscles. 2d. That of cells. 3d. The growth of the latter effected by vital imhibition, and 4th. Their compression and gradual conversion into minute lamillar or scales." And that these scales have spaces between them, being connected by a "translucent, gelatinous substance," which he states as having considerable "elasticity," and that the fluid secreted upon the surface of the vascular corium, contains the nuclei and corpuscles, from whence the scales, being the highest stage of development, originate.

aspect, and whose use he suggests as being rather designed for absorption than secretion.

The glands of Brunner are mostly found in the duodenum, and are seen to surround the intestine, in the form of a layer of white bodies, about the size of a hemp seed. They resemble the salivary glands, in consisting of lobules, having excretory ducts, which open into a common tube.

The glands of Peyer are found at the lower portion of the ileum, and are either collected in clusters, called (agminatæ,) or exist alone, and are termed (solitariæ.) The peculiarity of these glands consist in their having no excretory ducts, though they are found to contain mucus, and small vesicles or cells. They are seen to present themselves in circular or elliptical patches, with a slightly raised surface, and from being closed, their precise use still remains a problem.

The *common function* of mucous membrane, like the skin, consists in sensation, secretion, absorption, and in its capacity of self-protection from the action of external bodies. Its sensation, compared with the skin, is dull—its secretion and absorption are much more active, while its protective powers are remarkably great and astonishing, and are derived principally from the presence of a fluid, found every where belonging to this membrane, and called *mucus*. This fluid varies in its composition, as it is obtained from different points of the mucous surface, and in consequence of being mixed with so many other different fluids, its true character is consequently difficult to be arrived at. It is regarded, however, as a viscid fluid, "either colorless or slight yellow, transparent, or nearly so, incapable of mixing with water, and sinking in it." Its peculiar principle is termed *mucin*, which is made to consist of albumen, and, according to the analysis of Berzelius, besides a large quantity of water, contains the muriates of potassa and soda; lactate of soda, with animal matter, soda, albumen and animal matter, and phosphate of soda. In health this fluid is regarded as generally *alkaline*, though in disease often acid. Under the microscope it is found to contain epithelium scales, and granular corpuscles. Such is the nature of this fluid, destined to protect the general mucous surface.

With these remarks upon mucous membrane in general, we come now to inquire a little more particularly into the *mucous membrane of the mouth.* To the dentist, the mucous membrane of the mouth seems to involve the great anatomical specialty with which it is most desirable he should be thoroughly acquainted—but for whose thorough acquantance, he will soon find, that his knowledge must be further extended to the system at large, before he can expect to reap the full benefit to be derived by the practice of his profession.

The mucous membrane of the mouth forms one of the fundamental elements in the formation of the teeth, gums, lip, palate, tongue, tonsils, &c., and is as fundamentally concerned in the first stages of digestion and respiration—likewise in the function of taste, and with the still higher function of speech.

The mucous membrane lines the entire cavity of the mouth, forming loose folds or bridles at different points, as at the upper and lower lips, called (frenum labiæ)—at the under and anterior surface of the tongue (frenum linguæ)—at the englottis (frenum epiglotidis)—and on either side of the uvula, the lateral half arches of the palate constituting the fauces—and is traced continuous with the skin, as before mentioned, on the one hand, and found extending by a similar continuity of structure into the pharynx, larynx, eustachian tubes, the nostrils, the eye, and the maxillary and frontal sinuses.

The mouth, extending from the lips in front to the soft palate behind, and bounded laterally by the cheeks, has its mucous membrane presenting some very interesting and important modifications at different points. It has already been stated to have, with mucous membrane in general, an epithelium, a basement membrane, and a fibro-vascular structure or corium. The epithelium was also further stated to be of the squamous or tesselated variety. Now, upon the lips, this membrane by being exposed to the air, has its epithelium dryer and readily demonstrated. Its glands are numerous, and are found to be of the salivary variety and not muciparous follicles—they are called *labial salivary glands.* They are described as small spheroidal bodies of unequal size, numerous, situated between the mucous membrane and orbicularis muscle, and opening into the mouth by distinct orifices.

Upon the cheeks, the mucous membrane presents a somewhat similar character, and has its surface raised into projections by a similar series of glands, though of smaller size, lying upon the buccinator muscle, and called buccal glands. There are also two or three glands which open upon the buccal surface of the mouth by separate ducts opposite the last molar tooth, and are called molar glands. These are of the same salivary kind, and differ only by being of larger size, and in being situated between the masseter and buccinator muscles.

Upon the roof of the mouth forming the palatine processes of the superior maxillary bones, the mucous membrane, says M. Cruveilher, "is remarkable for its whitish color, for the thickness of its epithelium, especially in front—for the thickness and density of its chorion—for its close adhesion to the bones—and for the great number of orifices with which it is perforated, especially behind."

Upon the soft palate this membrane presents the two varieties, or rather the three varieties of epithelium—the upper surface or that which looks towards the posterior nostrils, having, according to Dr. Henle the *ciliated columnar epithelium*, like the mucous membrane of the nose—while the lower surface, or that which looks to the mouth, is of the squamous or tesselated variety common to the whole buccal cavity.

Upon the tongue the mucous membrane is remarkable for the roughness of its surface, the (almost cartilaginous) density and compactness of its chorion, of its attachment to muscles, of its being the seat principally of taste, and in its presenting numerous papillæ. The papillæ are seen upon the dorsum of the tongue, and are divided according to their form, into three classes, viz. the papillar *maximæ*, or *calyciform*, situated upon the back part of the tongue, arranged like the letter v reversed, and are the largest in size—2. The *mediæ*, or *fungiform*, next in size, occupy principally the sides and apex of the tongue, and have, says Mr. Harrison, a red color—the 3d class are called the *conical*, *filliform*, or *papillæ villosæ*—these are situated upon the greater portion of the dorsum of the tongue, are very numerous, present a whitish color, have their direction backwards, and give to the tongue its roughness or "brush like" appearance, as plainly seen by the naked eye, while these same papillæ in some of the lower animals, as the cow for example, are exceedingly long, hard, almost horny in their feel, resembling somewhat the rough surface of a rasp, and from their capability of being raised, and having both the forward and backward movement, are well suited as assistant masticating organs, in retaining and compressing the food, as well as giving it the backward direction towards the pharynx.

The papillæ, says Mr. Nasmyth, have a strong analogy in structure to the "pulps of the teeth"—and he continues, "the analogy does not rest here only, for just as the pulps are organs for the production of the teeth, so in some animals, as in birds, the papillæ of the tongue are the source of formation of horny appendages, which perform the offices of teeth, and bear a close relation to those organs." Capillary vessels and nervous filaments, bound together by "areolo-fibrous" tissue and covered by the squamous epithelial investment, compose these papillæ—and the valuable diagnosis which physicians derive from examining the tongue, are regarded as nothing more than the various changes which this epithelium of the papillæ undergo during disease.

Upon the gums, the mucous membrane is so thick and firm as to be called dental cartilage, but the absence of the corpuscles of Purkinje reject the idea of the cartilaginous character, and in lieu thereof, Mr. Nasmyth affirms that the gums "are made up of a mass of scales lying one on the surface of the other"—and that "the alveolar epithelium is thicker in proportion to the youth of the subject examined." The gums are very vascular and can be traced as continuous with the periosteum of the bones.

The last modification of the mucous membrane we have to notice, as connected with the mouth, is that of the teeth—and here, Mr. Goodsir and Mr. Nasmyth have not only shown the origin of the teeth to be from mucous membrane, but have further demonstrated with surprising minuteness, all the successive stages in their after development—changes so remarkable, that without a knowledge of the fact, one could scarcely credit the truth, that bodies so hard as the teeth, and apparently so dissimilar in every respect to the soft, delicate mucous membrane, could possibly have had any such parentage. The teeth were at one time classed among the bones, but now it is clearly demonstrated that the mucous membrane of the mouth first presents a groove; that, in this groove are seen little eminences, termed papillæ or tooth-germs, that these papillæ grow and are surrounded by a simultaneous growth of the walls of the groove, constituting first the follicle, and when closed, next the sac of the tooth—thus making the three stages of a tooth to consist in a papillary, follicular, and sacular, to which is added its eruptive, stage. Now Mr. Nasmyth makes the following observation in reference to this remarkable modification of the mucous membrane, as connected with the teeth, which we beg leave to quote: "The first trace, he says, of the future tooth, the dental papilla, is originally a part of the mucous membrane—subsequently, however, when the papilla is somewhat further advanced in growth, the two tissues are entirely distinct, the former being almost wholly composed of corpuscles or cells, the latter maintaining its fibrous character. At a later period the structure of the pulp is still more characteristically different from mucous membrane, while the internal surface of the capsule has become a serous rather than a mucous membrane. And, at this stage, its surface is invested with a very delicate layer of cells, totally different from those of epithelium."

With this account of the mucous membrane of the mouth, we will in addition add, that it receives upon its surface the salivary fluids, and is most liberally supplied with blood vessels and nerves.

The saliva comes from the six salivary glands, three on either side of the mouth, viz. the *parotid*, *submaxillary* and *sublingual*, the former supplying its fluid to the molar teeth, while the two latter supply those of the canine and incisor. The *blood vessels* for the lips and cheeks come from the coronary arteries of the facial, a branch of the external carotid, and the *buccal, infra-orbital, alveolar, mental* and *masseteric*—all branches of the internal maxillary, which also comes from the external carotid. The palate, gums, teeth, and tongue are supplied pretty much from the same source, the palatine arteries going to the palate, the superior coronary to the gums of the upper, and the sub-mental and sublingual to those of the lower jaw, while the superior, inferior dental, and infra-orbital go to the teeth. And the lingual, palatine, and inferior pharyngeal to the tongue. The veins correspond very closely with the arteries as to name and direction, and do not require further notice.

The nerves supplying all these various parts come principally from the 5th, 7th, 8th, and 9th pair.

The last point we propose to notice, is, the several *relations* of the mucous membrane of the mouth.

These may all be classed under three heads, viz. 1. The physical. 2. The organic, and 3. The mental.

The *physical*, are those which this membrane has with atmospheric air, our food and drinks, and the salivary fluids with which it is brought in contact, when introduced into the cavity of the mouth.

The air, it is known, has to pass through the mouth in its passage to the lungs during respiration. Now this air, in respiration, will carry to the lungs through the mouth, in contact with its lining mucous membrane, all the impurities with which it may be loaded from without, and return in expiration, in the same way, surcharged with deleterious properties which must be expelled from within, so that if the air should depart very materially from its natural healthy, or physiological condition, the mucous membrane of the mouth will be in danger of suffering in proportion to such departure, and the general condition of the system. Food, by remaining in the mouth, may undergo chemical decomposition, and develop corrosive agencies which may destroy the teeth and gums, as well as the other portions of this lining membrane of the mouth. The salivary fluids, though secreted alkaline in health, are, nevertheless, we are told, during fasting, acid, and then if we add to this the acid mucus of the mouth, which is said to be the case during disease; we have thus a combination of agencies capable of acting upon and injuring the best denture, while in one which is bad, ruin must be inevitable, if this state of things be not arrested by proper treatment.

The *organic relations* of the mucous membrane of the mouth are strikingly obvious, and most especially that portion covering the tongue, if examined when the general system is laboring under disease—for, here we find, as upon a tablet, that the stomach and liver, with other organs, report their complaints, and expect to find a sympathy of feeling in their behalf.

And here also it is, the physician invariably looks for his most valuable diagnosis, into the condition of distant organs, by simply inspecting the various appearances of the mucous epithelium as indicated upon the tongue—so also with the gums, the teeth, and every other portion of the mucous membrane of the mouth, each having more or less relation with the various organs of the body.

The brain, standing as the instrument and representation of the mind, constitute, what we have thought fit to term, the *mental relations* of the mucous membrane of the mouth.

It is well known that the mind has great influence in increasing, suspending, and altering the secretions—while on the other hand, tooth-ache may so irritate and fret the mind as to amount almost to partial insanity—and further, that if the teeth, palate, and other parts are wanting, the mind, having no medium of communication, is, so far as speech and articulation is concerned, silent and completely lost—and lastly, viewing the brain simply as an organ in sympathy with the mucous membrane of the mouth and the teeth, we see how these latter, in teething, will, through the fifth pair of nerves, so powerfully act upon the brain as, through it, to often throw the child into the most powerful convulsions, by expending its fury upon the muscular system.

